# Exploration of Key Factors in the Preparation of Highly Hydrophobic Silica Aerogel from Rice Husk Ash Assisted by Machine Learning

**DOI:** 10.3390/gels11010074

**Published:** 2025-01-17

**Authors:** Yun Deng, Ziyan Sha, Xingxing Wang, Ke Duan, Weijie Xue, Ian Beadham, Xiaolan Xiao, Changbo Zhang

**Affiliations:** 1 College of Environment and Ecology, Jiangnan University, Wuxi 214122, China; dengyun@jiangnan.edu.cn (Y.D.); 6231402018@stu.jiangnan.edu.cn (Z.S.); 6201403014@stu.jiangnan.edu.cn (X.W.); 6221402007@stu.jiangnan.edu.cn (K.D.); 8202101448@jiangnan.edu.cn (X.X.); 2 Key Laboratory of Original Agro-Environmental Pollution Prevention and Control (Ministry of Agriculture and Rural Affairs (MARA)), Tianjin Key Laboratory of Agro-Environment and Agro-Product Safety, Agro-Environmental Protection Institute, MARA, Tianjin 300191, China; 3 School of Pharmacy and Chemistry, Kingston University, London KT1 2EE, UK; i.beadham@kingston.ac.uk; 4 Institute of Future Food Technology, Jiangsu Jitri, Yixing 214200, China

**Keywords:** rice husk ash, silica aerogel, hydrophobicity, thermal stability

## Abstract

To expand the applications of hydrophobic silica aerogels derived from rice husk ash (HSA) through simple traditional methods (without adding special materials or processes), this paper employs machine learning to establish mathematical models to identify optimal conditions for extracting water glass and investigates how preparation conditions and heat treatment temperatures affect properties such as the porosity and hydrophobicity of HSA. The results indicate that the decision tree regression model provides the most accurate predictions for the extraction rate and modulus of water glass. Notably, the water contact angle of HSA produced using nitric acid as a catalyst can reach as high as 159.5°, classifying it as a superhydrophobic material. Additionally, while moderately increasing the concentration of the hydrophobic modifier enhances HSA’s hydrophobicity, it concurrently reduces its porosity. The HSA maintained hydrophobicity until 500 °C. The pore structure of HSA collapsed gradually with the increase in heat temperature. After treatment at 700 °C, HSA lost its hydrophobicity and the porous structure was severely damaged. Compared with silica aerogel using traditional silicon sources, the damage to pore structure and the crystallization occurred at lower temperatures, but the hydrophobicity remained at higher temperatures.

## 1. Introduction

As the largest byproduct in the rice processing process, approximately 120 million tons of rice husks are produced annually [1]. After the conversion into heat or energy by burning or gasification, rice husk ash, which accounts for 15~20% of rice husks, remains as a residual component and is not effectively used currently [2]. Rice husk ash contains 90–98% amorphous SiO_2_, which is easy to obtain and can be used to prepare silica aerogel [3]. This utilization not only reduces the environmental pollution caused by rice husk ash, but also allows for the high-value utilization of the ash. Additionally, this method can reduce the raw material costs of silica aerogel.

Nevertheless, the impurities in the solid waste are probably detrimental to certain properties of silica aerogel [4]. Tadjarodi et al. [5] prepared a silica aerogel from rice husk ash (HSA) by drying it under atmospheric pressure, resulting in a specific surface area of 315 m^2^/g, an average pore diameter of 9.8 nm, and a porosity of 85%. Akhter et al. [6] synthesized HSA with a specific surface area of 298 m^2^/g, a porosity of 79.5%, and a pore diameter of 9.2 nm by drying it under atmospheric pressure. Liang et al. [7] synthesized HSA with a specific surface area of 278.1 m^2^/g, a density of 0.133 g/cm^3^, and a hydrophobic angle of 128° by the freeze-drying method. All these reports only adopted the most conventional preparation processes, and the above properties of the prepared HSA were generally lower than those of silica aerogel prepared from high-quality silicon sources, the porosity and specific surface area of which are reported at about 85~99% and 500~1000 m^2^/g [8,9]. Therefore, researchers have to adopt the addition of materials or process steps to improve the performance of HSA [10,11,12,13,14]. Although these measures have indeed improved its properties, they have gone against the original intention of using rice husk as a raw material to reduce costs and environmental pollution.

Since the thermal insulation, sound insulation, and other properties of silica aerogel are generally closely related to its porosity, and the larger the porosity is, the lower the thermal conductivity will be [15]. Therefore, HSA prepared by simple conventional methods (without adding special materials or processes) generally has no advantage in these application domains. Our previous study proved that HSA prepared using a simple conventional protocol could be utilized as a filler to enhance the corrosion resistance of epoxy coating [16]. The anti-corrosion performance was superior to that reported for certain conventional fillers in the literature, primarily due to its hydrophobicity, which remains unaffected by the use of a low-quality silicon source. Hydrophobicity plays a pivotal role during the Atmospheric Pressure Drying (APD) process as well. In comparison to supercritical drying and freeze-drying, the APD method stands out for its simplicity, safety, low cost, and ease of scale-up. However, in the course of APD, the hydrogen bonds that form between the hydroxyl groups on the SiO_2_ surface and water molecules get disrupted. Concurrently, the tensile stress induced by the capillary force due to water evaporation can inflict damage on the gel network skeleton, leading to the collapse of the pore structure. Hence, the possession of favorable hydrophobic properties, both at ambient and elevated temperatures, is of great significance for facilitating the application of APD [17]. Moreover, the retention of hydrophobicity at high temperatures broadens the temperature range within which the aerogel can be utilized, encompassing high-temperature environments [18].

Furthermore, the extraction of water glass from rice husk ash serves as a crucial starting point in the production of HSA. A high extraction rate of water glass helps to boost the economic viability of HSA, and an appropriate modulus of water glass plays a pivotal role in determining the properties of HSA. On the other hand, the extraction process of water glass involves multiple process parameters that intricately influence both the extraction rate and the modulus. Among the various experimental design methods used to identify the optimal parameters, the orthogonal experimental design and the response surface method have been the go-to choices. Although these traditional methods can replace comprehensive global experiments with more manageable local ones, thus reducing the experimental workload, they often fall short of delivering truly optimal results. There is a considerable risk that the outcomes obtained might deviate substantially from the global best combination. In contrast, by employing machine learning techniques to zero in on the optimal process parameters, we can effectively bridge this gap and minimize the deviation. This innovative approach offers a more accurate and efficient way to optimize the extraction process, ensuring that we get the most out of rice husk ash and produce high-quality HSA.

Therefore, this paper utilized machine learning methods to determine the optimal process parameters for water glass extraction from rice husk ash. Subsequently, using water glass as the silicon source, HSA was prepared using a simple conventional protocol and ambient drying method. The influence of the preparation conditions on the properties of the aerogel was investigated, with a particular focus on the hydrophobicity and high-temperature stability of the aerogel. This research aims to support the expansion of the application fields of low-cost HSA, such as in areas with relatively low porosity requirements and high hydrophobicity requirements, like fillers in anti-corrosion coatings.

## 2. Results and Discussion

### 2.1. Extraction of Water Glass

**Figure 1:**
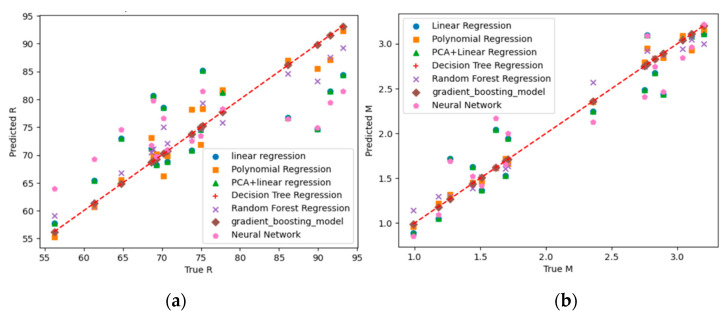
Training results of models: (**a**) for R; (**b**) for M.

We established a linear regression model (LR), polynomial regression model (PR, degree = 2), principal component analysis + linear regression model (PCA), decision tree regression model (DTR), random forest regression model (RFR), gradient boosting model (GB), and neural network model (NNM). The relationships between the predicted values and the experimental values are shown in Figure 1 and the values of the evaluation indicators of these models are listed in Table 1.

It is obvious that the predicted values of the DTR and GB fit very well with the experimental values. Using DTR to predict the conditions that maximize the Z value, the results are C = 1.1 mol/L, r = 4.5, T = 98 °C, and t = 3.65 h, and Z is 176.6. Using these conditions, the experimental R = 94.4%, M = 2.8, and the corresponding Z is 174.5.

### 2.2. Different Acid Catalysis

**Figure 2:**
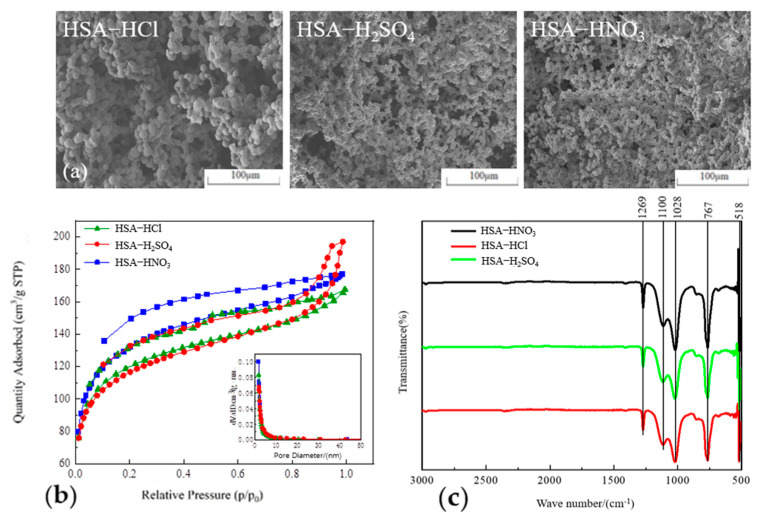
(**a**) Microscopic morphology; (**b**) N_2_ adsorption–desorption isotherms and pore diameter distribution; and (**c**) FTIR spectrum of HSA catalyzed by different acids.

Based on the SEM images of HSA prepared with different acids (Figure 2a), all samples exhibit a typical three-dimensional network structure, and there are a few large pores in the skeletal structure. The network structure is relatively complete, and the particle sizes are uniform. The N_2_ adsorption–desorption curves (Figure 2b) are in good agreement with Type IV isotherms, according to the classification of the International Union of Pure and Applied Chemistry [19], and the N_2_ adsorption capacity is from 167 to 197 cm^3^/g. The pore sizes of the samples are similar, mainly distributed around 2.5 nm, presenting a typical mesoporous structure (2–50 nm). The average pore sizes in HSA ranged 2.2–2.8 nm, whilst the specific surface areas ranged 430.4–487.1 m^2^/g, respectively. Among them, HSA-H_2_SO_4_ has the largest N_2_ adsorption capacity, but HSA-HNO_3_ has the largest specific surface area and the smallest average pore size. Therefore, nitric acid was used as a catalyst in the subsequent experiments.

In the FTIR spectrum of HSA (Figure 2c), the three curves are basically the same, indicating that different acid catalysts have no significant impact on the functional groups of HSA. The absorption peak at 518 cm^−1^ corresponds to the bending vibration of Si-O-Si, while the peak at 767 cm^−1^ is associated with the symmetric stretching vibration of the Si-O-Si bond. The absorption peaks in the vicinity of 1100 cm^−1^ and 1028 cm^−1^ are attributed to the asymmetric stretching vibration of the Si-O-Si bond, signifying the presence of a substantial quantity of Si-O-Si bonds within the aerogel [17]. The absorption peak at 1269 cm^−1^ pertains to the vibration of Si-CH_3_, indicating that the hydrophilic -OH groups on the surface of the HSA have been supplanted by hydrophobic -CH_3_ groups after modification. Correspondingly, the HSA exhibited high hydrophobicity with water contact angles exceeding 138.3°. Notably, the water contact angle of HSA-HNO_3_ was as high as 159.5°, qualifying it as a superhydrophobic material (water contact angle > 150°) [20].

**Figure 3:**
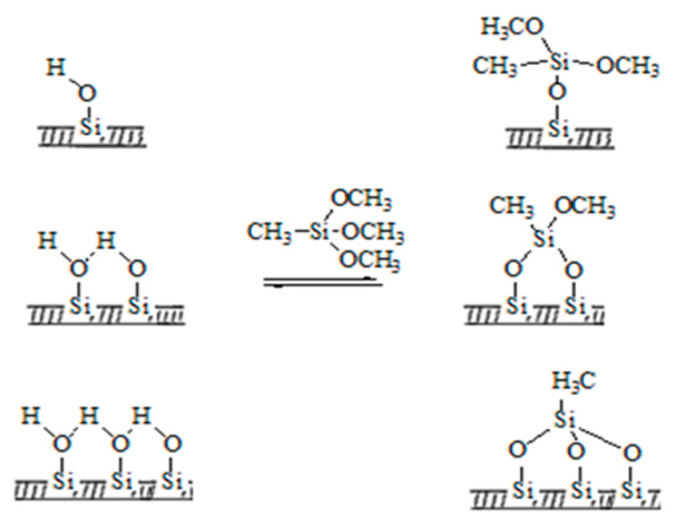
Possible reactions during the process of hydrophobic modification.

It is generally believed that under anhydrous conditions, the hydroxyl groups on SiO_2_ react with the hydrolysis group of MTMS to form Si-O-Si bonds, and the introduced hydrophobic group, methyl, can improve the hydrophobicity [21]. Since MTMS has three hydrolysis groups, it can be inferred that the following reactions may have occurred during the process of hydrophobic modification (Figure 3):

Notably, no peak was observed around 1635~1641 cm^−1^ in this study, a peak that typically appears in the FTIR spectrum of silica aerogel and is associated with -OH groups. This absence suggests that all -OH groups have interacted with MTMS, which accounts for the high hydrophobicity of these samples.

The influence of the concentration of the modifier MTMS on the properties of HSA was investigated. As MTMS concentration increased, the specific surface area, pore volume, and porosity of the aerogel gradually decreased, while the hydrophobicity slightly increased. There was little difference in the properties of HSA-0.2 and HSA-0.4. However, the specific surface area of HSA-0.8 dropped from 507.25 m^2^/g to 402.87 m^2^/g, the average pore diameter decreased from 5.89 nm to 2.95 nm, and the pore volume reduced from 0.75 cm^3^/g to 0.30 cm^3^/g (Table 2). This may be due to the fact that when the concentration of MTMS is relatively high, some methyl groups do not react with the hydroxyl groups on the surface of the aerogel and accumulate in the pore structure to some extent, resulting in an increase in density and a decrease in porosity [22,23].

**Figure 4:**
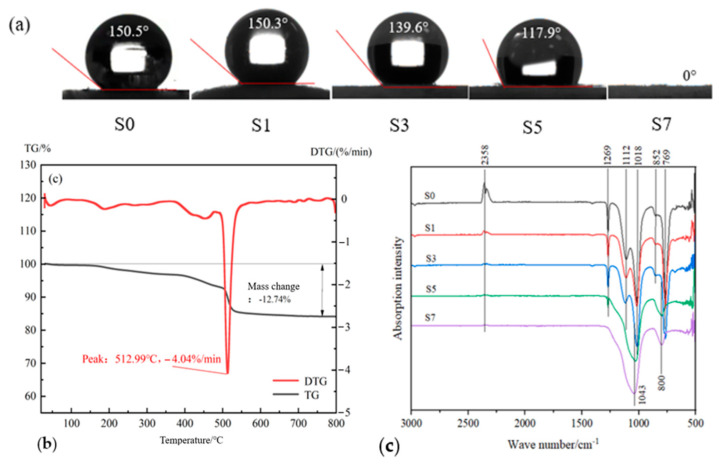
(**a**) Water contact angles; (**b**) TG and DTG curves; (**c**) FTIR spectra of HSA samples.

The aerogel without heat treatment had a water contact angle of 150.5° (Figure 4a) and can be classified as a superhydrophobic material (water contact angle > 150°). The hydrophobicity is mainly due to the methyl groups grafted during the surface modification, which can be confirmed from the peak at 1269 cm^−1^ in the S0 infrared spectrum (Figure 4c), i.e., the bending vibration peak of C-H. After treatment at 100 °C, the aerogel sample remains superhydrophobic with a water contact angle of 150.3°. Correspondingly, the C-H peak in the S1 infrared spectrum does not change significantly. After treatment at 300 °C and 500 °C, the water contact angle decreased to 139.6° and 117.9°, respectively, indicating that the sample was still hydrophobic (water contact angle > 90°). Correspondingly, the C-H peak in the infrared spectrum decreased, but it was still observed. The water contact angle of HSA treated at 700 °C was 0°, indicating that the hydrophobicity of HSA is completely lost at this temperature, and it becomes a hydrophilic material. This is attributed to the complete decomposition of the grafted methyl groups, which is in line with the vanishing of the peak at 1269 cm^−1^ in the S7 infrared spectrum (Figure 4c). Concurrently, the collapse of the porous structure of HSA leads to alterations in surface roughness, and this, in turn, can possibly modify its hydrophobicity as well [24]. The standard deviation of the contact angle value is 0.8 degrees.

In addition, in the FTIR spectra of S0 (Figure 4c), the peaks at 852 cm^−1^ and 769 cm^−1^ are the symmetric stretching vibration peaks of Si-O. The peaks at 1112 cm^−1^ and 1018 cm^−1^ are the antisymmetric stretching vibration peaks of Si-O. After heat treatment, the positions of the Si-O peaks in S1 and S3 are the same as those in S0, but the peak intensities are weakened to a certain extent. The results show that temperatures of 100 °C and 300 °C do not change the chemical bond connected to Si-O, but cause partial fracture of the Si-O bond. In S5 and S7, the Si-O peaks were further reduced, so that the sub-peaks were not obvious, indicating that more Si-O bonds were broken. The peak positions shifted compared with S0, indicating that the chemical bond connected to Si-O was changed, which may suggest that the C-Si bond was broken.

The peak at 2358 cm^−1^ may be due to a C=O bond, which was possibly generated by the oxidation of methyl during the drying process, and/or the adsorbed CO_2_, or carbonates formed by the reaction of CO_2_ with impurities [25]. After heat treatment, the peak decreased with the increase in heating temperature, indicating that the quantity of carbonyl decreased with heating temperature.

**Figure 5:**
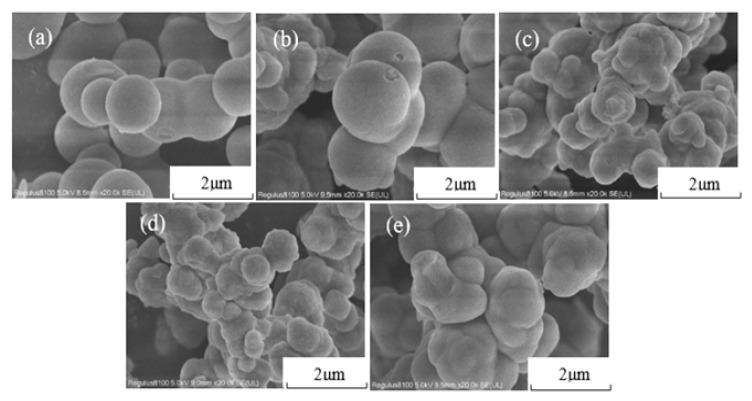
SEM images of HSA: (**a**) S0; (**b**) S1; (**c**) S3; (**d**) S5; (**e**) S7.

The effect of heat treatment on morphology was characterized by SEM (Figure 5). There is no significant change in S1 compared to S0. With the increase in heat treatment temperature, the particles inside the aerogel gradually agglomerated, the sample became denser, and the pores between the secondary accumulated particles decreased. After treatment at 700 °C, the secondary particles of S7 merged to form larger particles. The sintering is due to the atomic diffusion intensifying with the increase in heat treatment temperature, and the inter-particle contact changes from point contact to surface contact. As a consequence, the inter-particle contact area increases and the aperture gaps reduce. The closure of connected pores results in the consolidation of larger particles and the densification of material [26]. Nevertheless, the network structure of all the samples can clearly be seen to be similar to the untreated sample, and the porous structure is also almost maintained.

**Figure 6:**
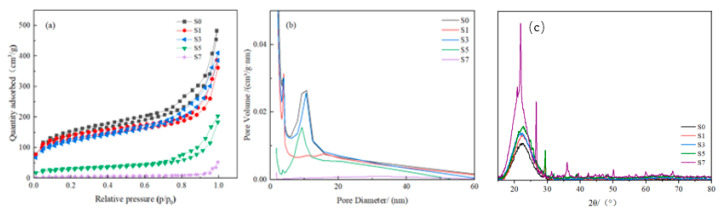
(**a**) N_2_ adsorption–desorption isotherms; (**b**) pore size distribution; and (**c**) XRD patterns of HSA.

The N_2_ adsorption–desorption isotherms of the HSAs are shown in Figure 6a. After heat treatment, the N_2_ adsorption capacity decreased with the increase in heating temperature. S3 still had a large adsorption capacity. S5 showed a significant decrease in adsorption capacity, and S7 showed almost no adsorption capacity. All the samples have type IV curves with a H_3_ hysteresis loop [27]. This indicates that all the samples exhibited flaky particles with an irregular mesoporous structure. In other studies, the N_2_ adsorption–desorption isotherms of silica aerogel usually exhibited type IV curves, but with H_1_, H_2_, or H_3_ types of hysteresis [23,28].

The pore size distributions of the samples are presented in Figure 6b. For S0 and S3, two peaks at pore diameters of ∼6 nm and ∼10 nm were observed. S1 exhibited exception, probably due to the instability of rice husk ash composition. The two peaks of S5, especially the peak at pore diameters of ∼6 nm, decreased obviously. In S7, no peak was observed. In S5 and S7, the secondary particles coalesced (Figure 5), resulting in the diminution of the pores surrounded by these particles. At the same time, other pores collapsed and larger pores formed. Correspondingly, the average pore diameter and tap density increased, whilst BET surface area and BET measured pore volume decreased (Table 2). The BET surface area of S5 and S7 decreased by 79.6% and 96.5% compared to S0, respectively. But in the study of He et al. [29], the BET surface area decreased only by 31.7% and 38.9%, respectively, after heat treatment at 500 °C and 600 °C. Smaller pores and lower porosity may reduce the penetration of water molecules, thereby increasing hydrophobicity [30].

The thermal stability of HSA is relatively poor compared to aerogels prepared from conventional silica sources in the literature, such as ethyl orthosilicate. For example, in the study of Cui et al., the BET surface area only decreased by 8.65% after heat treatment at 500 °C [31]. In the study of Liao et al., the aerogel maintained its amorphous structure after heat treatment at 1000 °C [8]. This may be due to the presence of more impurities in HSA, such as Al^3+^, K^+^, Ca^2+^, Mg^2+^, and other impurities [9].

In the XRD patterns of S0, S1, and S3, there is only a broad peak at 2ϴ ≈ 21.5° (Figure 6c), suggesting that silica aerogel is an amorphous and non-crystalline material [21,23,32]. In the XRD pattern of S5, there is a protuberance on the broad peak shoulder, suggesting that some crystals formed at 500 °C, and the possible crystal phases include β-Quartz and coesite. β-Quartz characteristic peaks are at about 2ϴ ≈ 22.5° and 24.8°, and coesite characteristic peaks are at about 2ϴ ≈ 21.8° and 25.9° [33]. The narrow and high diffraction peak in the XRD pattern of S7 indicates that the silica changes from amorphous to crystalline form at 700 °C. The crystalline phases might consist of α-Quartz and/or β-Quartz, both of which possess characteristic peaks at approximately 20.9°, 26.6°, 36.7°, and 42.3°, and are likely to not be readily distinguishable [33]. In fact, it has been confirmed by many research reports that silica aerogel using traditional silica sources can retain its non-crystalline structure, even when heated to 1200 °C and 1300 °C [25,34,35]. At temperatures higher than 1200 °C and 1300 °C, crystallization occurred due to the high OH content present in the material [36]. Since no OH peak was observed in this study, the crystallization is probably because the heteroatoms in straw ash could catalyze the reaction or react directly with silica at lower temperatures (500 °C) to form crystals.

## 3. Conclusions

The crucial factors in the preparation of highly hydrophobic HSA involve the extraction of water glass, the choice of acid catalysts, and the hydrophobic modification process. With the help of machine learning, the optimal conditions for extracting water glass from rice husk ash, which ensure a higher extraction rate and an appropriate modulus, can be obtained through fewer experiments. It has been discovered that the decision tree regression model can offer the most precise predictions for the extraction rate and modulus of water glass. Employing nitric acid as a catalyst and opting for an appropriate modifier concentration can further enhance the hydrophobicity.

As the heat treatment temperature rises, the pore structure and hydrophobic properties of HSA gradually deteriorate. Concurrently, the specific surface area and pore volume decrease, being particularly prominent at 500 °C and 700 °C. The hydrophobicity of HSA can be maintained up to 500 °C. However, after being treated at 700 °C, the aerogel loses its hydrophobicity, predominantly because of the decomposition of hydrophobic groups, and its porous structure suffers severe damage.

Compared with silica gel made from common silica sources, the degradation of the pore structure and the onset of crystallization already occur at relatively low temperatures. The impurities present in rice husk ash may result in unstable sample performance and reduced heat resistance.

## 4. Materials and Methods

### 4.1. Materials

Hydrochloric acid (HCl, 36–38%), nitric acid (HNO_3_, 65–68%), sodium hydroxide, anhydrous ethanol (EtOH, 99.7%), and n-hexane (n-hexane, 97%) were purchased from Sinopharm Chemical Reagent Co., Ltd. (NO. 52, Ningbo Road, Shanghai, China) Methyl trimethoxysilane (MTMS) was purchased from Aladdin Biochemical Reagent Co., Ltd. (No. 809, Chuhua Branch Road, Fengxian District, Shanghai, China)

Rice husks were purchased from Only Produce Store and then were calcined in a muffle furnace at 600 °C for 4 h to obtain rice husk ash. The compositions (wt%) of the rice husk ash samples were determined using an X-ray fluorescence spectrometer, and are shown in Table 3.

### 4.2. Extraction of Na_2_SiO_3_

Rice husk ash was mixed with NaOH solution at a certain ratio and the mixture was heated at a certain temperature for a certain time and then filtrated to obtain water glass (Na_2_O·nSiO_2_). Tests were conducted under different conditions, with variations in the concentration of the NaOH solution (C), the ratio of the NaOH solution to rice husk ash (r), the reaction temperature (T), and the reaction time (t). The dissolution rate of SiO_2_ (R) in rice husk ash and the modulus of water glass (M) were measured.

Python language was used to write code to establish mathematical models of the relationship between the experimental conditions and R and M. Taking the coefficient of determination (R^2^), root mean square error (RMSE), and mean absolute error (MAE) as the evaluation indicators for selecting the optimal model, the score of the extraction process (Z) was calculated using Formula (1) [37]:

Z = 100R + (100 − 66.23 × |M − 2.5|) (1)

Then, we used the model to predict the process parameters with the highest Z value for extracting water glass for standby use.

### 4.3. Preparation of HSA

The pH of the above solution was adjusted to 9 using a 0.5 mol/L acid solution. The HSA samples with hydrochloric acid, sulfuric acid, and nitric acid were denoted as HSA-HCl, HSA-H_2_SO_4_, and HSA-HNO_3_, respectively. After the wet gel was formed and aged at room temperature for 12 h, it was immersed in ethanol at 40 °C for 12 h. Equal molar amounts of MTMS and n-hexane were added to ethanol to be used as a surface modifier, with three variations being used. The three concentrations of MTMS were 0.2, 0.4, and 0.6 mol/L for HSA denoted as HSA-0.2; 0.4, 0.6, and 0.8 mol/L for HSA denoted as HSA-0.4; and 0.6, 0.8, and 1 mol/L for HSA denoted as HSA-0.8, respectively. Finally, the wet silica gel was cleaned with n-hexane, dried at 37.5 °C for 24 h, and then dried at 65 °C for 24 h to obtain HSA.

The HSA that was prepared using the optimized conditions was put in a muffle furnace and treated at 100 °C, 300 °C, 500 °C, and 700 °C for 4 h, respectively, named as S1, S3, S5, and S7. The sample without heat treatment, used as the control, was denoted as S0. The physical and chemical properties of the samples were determined.

### 4.4. Characterization of Samples

The modulus of water glass was determined using the method in reference [38]. The surface morphology of the samples was analyzed by SEM (Regulus 8100, Hitachi, Tokyo, Japan). The specific surface area (S_BET_) and average pore size (D_pore_) of the samples were calculated using the BET (Brunauer–Emmett–Teller) method with a fully automated specific surface and porosity analyzer (HSAP 2460, Micromeritics, Norcross, GA, USA). The functional groups of the samples were analyzed with FTIR tests (Nexus 470, Nicolet, Waltham, MA, USA) in the range of 4000 cm^−1^–400 cm^−1^, with a resolution of 4 cm^−1^. The hydrophobicity of the samples was characterized by the contact angle of the water drops on the surface of the aerogel (θ_w_), which was measured with a static drop contact angle/surface tension tester (OCA20, Dataphysics Instruments GmbH, Filderstadt, Germany, Purchasing in Beijing). The phase of the samples was measured using an X-ray diffractometer (D2 Phaser, Bruker, Berlin, Germany, Purchasing in Shanghai). The test target was copper, with a scanning range of 10–80° and a scanning speed of 2°/min. The tap density was measured manually. A certain amount of RHHSA was put in a cylinder and the powder volume was read after compaction. The tap density (*ρ*_m_) and porosity (P) were calculated according to the following formulas:

*ρ*_m_ = m/V (2)

P = (1 − *ρ*_m_/*ρ*_A_) × 100% (3)

where m and V are the weight and volume of the sample, respectively, and *ρ*_A_ is the theoretical density of the porous silica (2.2 g/cm^3^).

The thermal stability was analyzed using a thermogravimetric analyzer (STA 449 F5, Netzsch, Selb, German) with a heating rate set at 10 °C/min and a heating range of 50–800 °C.

## Figures and Tables

**Table 1:** Evaluation indicators of the models.

Property	Indicator	LR	PR	PCA	DTR	RFR	GB	NNM
R	R^2^	0.47	0.92	0.47	1	0.94	0.9999	0.52
	RMSE	7.58	2.89	7.58	0.0	2.51	0.02	7.26
	MAE	6.21	2.41	6.21	0.0	2.11	0.01	5.94
M	R^2^	0.89	0.991	0.89	1	0.98	0.9999	0.88
	RMSE	0.25	0.07	0.25	0.0	0.11	0.0	0.26
	MAE	0.21	0.05	0.21	0.0	0.08	0.0	0.21

**Table 2:** Textural properties of HSA.

Samples	*ρ*_m_ (g/cm^3^)	P (%)	S_BET_ (m^2^/g)	D_pore_ (nm)	V_pore_ (cm^3^/g)
S0	0.13	94	507.25	5.9	0.64
S1	0.15	93	470.64	5.1	0.59
S3	0.18	92	435.79	5.8	0.63
S5	0.28	86	103.53	12.2	0.31
S7	0.42	81	17.68	18.3	0.08

**Table 3:** Ash compositions (wt%) of the rice husk samples.

SiO_2_	Al_2_O_3_	K_2_O	CaO	MgO	Na_2_O
98.548	0.464	0.129	0.104	0.086	0.053

## Data Availability

The original contributions presented in this study are included in the article. Further inquiries can be directed to the corresponding authors.
